# A multitask bi-directional RNN model for named entity recognition on Chinese electronic medical records

**DOI:** 10.1186/s12859-018-2467-9

**Published:** 2018-12-28

**Authors:** Shanta Chowdhury, Xishuang Dong, Lijun Qian, Xiangfang Li, Yi Guan, Jinfeng Yang, Qiubin Yu

**Affiliations:** 1 Center of Excellence in Research and Education for Big Military Data Intelligence (CREDIT), Department of Electrical and Computer Engineering, Prairie View A&M University, Texas A&M University System, Prairie View, Texas, 77446 USA; 2 Schools of Computer Science and Technology, Harbin Institute of Technology, Harbin, China; 3 Schools of Software, Harbin University of Science and Technology, Harbin, China; 4 Second Affiliated Hospital of Harbin Medical University, Harbin, China

**Keywords:** Recurrent neural network, Multitask learning, Word embedding, Parts-of-speech tagging, Named entity recognition, Electronic medical records

## Abstract

**Background:**

Electronic Medical Record (EMR) comprises patients’ medical information gathered by medical stuff for providing better health care. Named Entity Recognition (NER) is a sub-field of information extraction aimed at identifying specific entity terms such as disease, test, symptom, genes etc. NER can be a relief for healthcare providers and medical specialists to extract useful information automatically and avoid unnecessary and unrelated information in EMR. However, limited resources of available EMR pose a great challenge for mining entity terms. Therefore, a multitask bi-directional RNN model is proposed here as a potential solution of data augmentation to enhance NER performance with limited data.

**Methods:**

A multitask bi-directional RNN model is proposed for extracting entity terms from Chinese EMR. The proposed model can be divided into a shared layer and a task specific layer. Firstly, vector representation of each word is obtained as a concatenation of word embedding and character embedding. Then Bi-directional RNN is used to extract context information from sentence. After that, all these layers are shared by two different task layers, namely the parts-of-speech tagging task layer and the named entity recognition task layer. These two tasks layers are trained alternatively so that the knowledge learned from named entity recognition task can be enhanced by the knowledge gained from parts-of-speech tagging task.

**Results:**

The performance of our proposed model has been evaluated in terms of micro average F-score, macro average F-score and accuracy. It is observed that the proposed model outperforms the baseline model in all cases. For instance, experimental results conducted on the discharge summaries show that the micro average F-score and the macro average F-score are improved by 2.41% point and 4.16% point, respectively, and the overall accuracy is improved by 5.66% point.

**Conclusions:**

In this paper, a novel multitask bi-directional RNN model is proposed for improving the performance of named entity recognition in EMR. Evaluation results using real datasets demonstrate the effectiveness of the proposed model.

## Background

Electronic Medical Record (EMR) [1], a digital version of storing patients’ medical history in textual format, has shaped our medical domain in such a promising way that can gather all information into a place for healthcare providers. It comprises both structured and unstructured data that consists of patients’ health condition and information such as symptoms, medication, disease, progress notes, and discharge summaries. EMR facilitates medical specialists and providers to track digital information and monitor them for patients’ regular check-up. It can also provide healthcare suggestions to patients even they live in a remote area. Moreover, when a patient switches to a new healthcare provider, the provider can easily obtain patients’ medical history and current health condition by studying patient’s EMR. Therefore, information extraction [2] from EMR is one of the most important tasks in medical domain. The intent of information extraction system is to identify and connect the related information and organize them in such a way that can help people to draw conclusions from it, and by avoiding the unnecessary and unrelated information.

To extract information like entity recognition from EMR is labor intensive and time consuming. Although there are many developed models for extraction of entity terms from textual documents, adopting these models for the purpose of medical entity recognition from EMR has been demonstrated as a challenging task, because most of the EMRs are hastily written and incompatible to preprocess [2]. Moreover, incomplete syntax, numerous abbreviation, units after numerical values make the recognition task even more complicated [3]. Standard Natural Language Processing (NLP) tools cannot perform efficiently when they are applied on EMR, since the entity terms of standard NLP is not designed for medical domain. Therefore, it is necessary to develop effective method to perform entity recognition from EMR.

In recent years, various deep learning based methods have been developed for Named Entity Recognition (NER) [4] from EMR. Convolutional Neural Network (CNN) model is used for NER by using data mining to enhance the performance [5]. Zao et al. [6] proposed multiple label CNN based disease NER architecture by capturing correlation between adjacent labels. Dong et al. [7] developed multiclass classification based CNN for mining medical entity types from Chinese EMR.

Most recently, Recurrent Neural Network (RNN) such as Long Short-Term Memory (LSTM) is taking prominent place in NER due to its ability of dependency building in neighboring words. A hybrid LSTM-CNN is proposed in [8]. The authors used CNN to extract the features and fed them to LSTM model for recognizing entity types from CoNLL2003 dataset. Wang et al. [9] studied bi-directional LSTM architecture and concluded that this model is very effective for predicting sequential data. Moreover, the performance of the model is not based on language dependency. Simon et al. [10] and Vinayak et al. [11] used bi-directional RNN model on their Swedish EMR and Hindi dataset, respectively. In each case, the model shows better performance comparing to the state-of-the-art model. Similarly, the approach of using bi-directional RNN with LSTM cell has proven to perform well in extracting named entity recognition task [12].

**Fig. 1:**
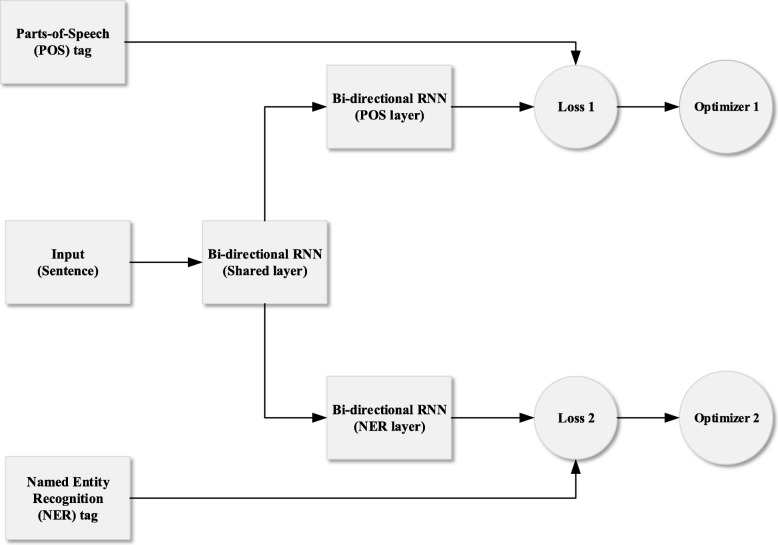
Framework of the proposed multitask bi-directional RNN model for NER

In general, large corpus dataset is required to train deep learning models. However, there are limited number of corpus in many existing datasets that hinders the development of NER. Moreover, building labeled Chinese EMR data faces many challenges [13], and most organizations do not want to share their data publicly as the data contains private information of patients. In order to address this challenge, a multitask bi-directional RNN model is proposed in this work for extracting entity terms from Chinese EMR. It is motivated by the observation that the performance of multitask learning model is much better comparing to individual learning approach when there is limited corpus dataset [14]. The framework of the proposed multitask bi-directional RNN model for NER is given in Fig. 1.

## Methods

**Fig. 2:**
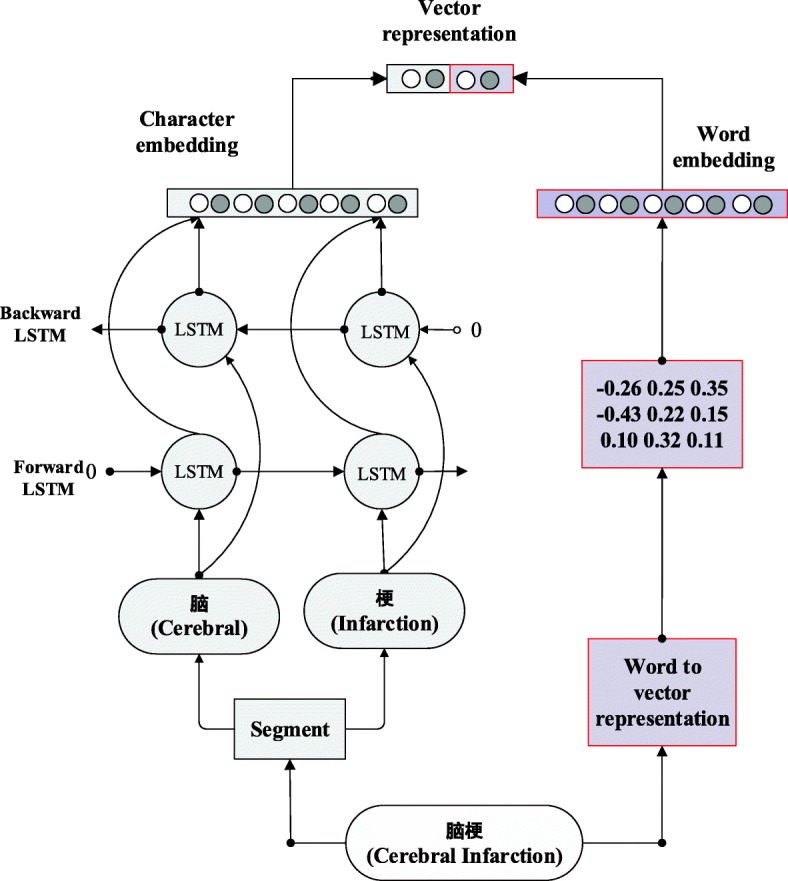
Vector Representation as concatenation of word embeddings and character embeddings. Vector representation of each word is presented as concatenation of word embeddings and character embeddings. The flow of word embedding is highlighted by red shaded box and character embedding is highlighted by white shaded box

In this work, a multitask bi-directional RNN model is proposed for extracting entity terms from Chinese EMR. The proposed model can be divided into two parts: shared layer and task specific layer, see Fig. 1. Specifically, vector representation of each word is a concatenation of word embedding and character embedding in the proposed model, see Fig. 2. Bi-directional RNN is used to extract context information from sentence. Then all these layers are shared by two different task layers, namely the parts-of-speech tagging task layer and the named entity recognition task layer. These two tasks layers are trained alternatively so that the knowledge learned from named entity recognition task can be enhanced by the knowledge gained from parts-of-speech tagging task.

RNN [15] is an artificial neural network which can capture previous word information of a sequence in its memory. It computes each word of input sequence (*x*_1_, *x*_2_, ⋯, *x*_*n*_) and transforms it into a vector form (*y*_*t*_) by using the following equations:

1 $$ h_{t}= H(U_{xh}x_{t}+U_{hh}h_{t-1}+b_{h}).  $$

2 $$ y_{t} = U_{hy}h_{t}+b_{y}.  $$

where *U*_*xh*_, *U*_*hh*_, *U*_*hy*_ denote the weight matrices of input-hidden, hidden-hidden and hidden-output processes, respectively. *h*_*t*_ is the vector of hidden states that capture the information from current input *x*_*t*_ and the previous hidden state *h*_*t*−1_.

Here the bi-directional RNN is used to exploit both past and future context, where forward hidden states compute forward hidden sequence while backward hidden states compute backward hidden sequence. The output *y*_*t*_ is generated by integrating the two hidden states. In this work, we use a special form of bi-directional RNN, the bi-directional RNN with LSTM cell [16]. LSTM is a special kind of RNN where hidden states are replaced by memory cells to capture long term dependent contextual phrase. The computation of LSTM is quite similar to RNN except for the hidden units, and it is given below:

3 $$ i_{t}= \sigma\left(U_{xi}x_{t}+U_{hi}h_{t-1}+ U_{ci}c_{t-1}+b_{i}\right).  $$

4 $$ g_{t}= \sigma\left(U_{xg}x_{t}+U_{hg}h_{t-1}+ U_{ci}c_{t-1}+b_{g}\right).  $$

5 $$ c_{t} = g_{t} c_{t-1}+i_{t} \tanh\left(U_{xc}x_{t}+U_{hc}h_{t-1}+b_{c}\right).  $$

6 $$ y_{t}= \sigma\left(U_{xy}x_{t}+U_{hy}h_{t-1}+U_{cy}c_{t}+b_{y}\right).  $$

7 $$ h_{t}= y_{t} \tanh(c_{t}).  $$

where *i*, *g*, *c*, *o* and *σ* are the input gate, forget gate, cell activation vector, output gate, and logistic sigmoid function of LSTM cell, respectively. These gates and activation functions soothe LSTM to avoid the limitation of vanishing gradients by storing long term dependencies terms of a sequence.

**Fig. 3:**
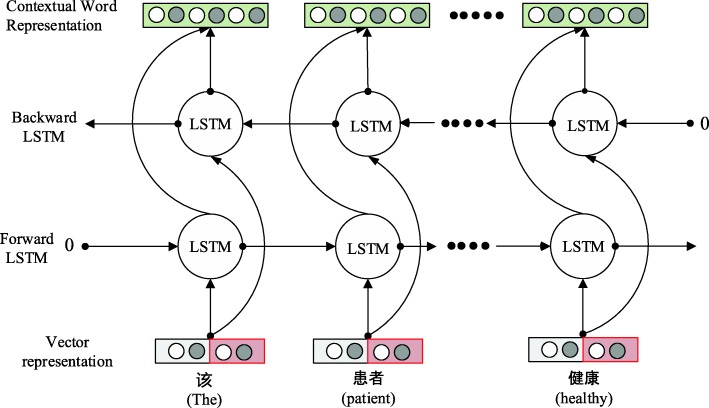
Contextual word representation from vector representation. To extract relevant context information from sentence, bi-directional RNN with LSTM cell is used to extract information from a vector associated with word embedding (red shaded box) and character embedding (white shaded box) to form contextual word representation (green shaded box)

The shared layer contains two consecutive parts, illustrated by Figs. 2 and 3. In Fig. 2, each word is represented by a vector developed by Mikolov [17]. The vector is built as a concatenation of word embeddings [18] and character embeddings. Bi-directional RNN with LSTM cell is used to extract features at the character level and represent the features as character embeddings. Word embedding is achieved by word to vector representation. Character embeddings and word embeddings are then combined to represent each word in a vector representation. In Fig. 3, another bi-directional RNN with LSTM cell is used to extract context information from text sequence. Then the outputs (contextual word representations) are shared by two different bi-directional RNN with LSTM cell for two different tasks: parts-of-speech tagging and named entity recognition. These two task layers are trained alternatively so that knowledge from parts-of-tagging task can be used to improve the performance of named entity recognition task [19]. The detailed settings of the proposed model is shown in Table 1.

**Table 1:** The proposed network architecture

Name	Description
Input	Sentences in EMR
Word Embedding	Mikolov model
Character Embedding Layer	150 LSTM cells for each hidden layer,
	one forward hidden layer and one backward hidden layer,
	Dropout = 0.5
Parts-of-speech tag (POS) layer	150 LSTM cells for each hidden layer,
	one forward hidden layer and one backward hidden layer,
	Dropout = 0.5
Named Entity recognition (NER) Layer	150 LSTM cells for each hidden layer,
	one forward hidden layer and one backward hidden layer,
	Dropout = 0.5
Output	Softmax

## Results

### Dataset details

**Fig. 4:**
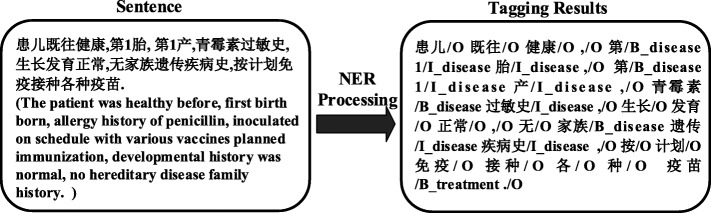
Tagging results on Chinese EMR [7]

The EMR dataset used in our experiment was collected from the departments of the Second Affiliated Hospital of Harbin Medical University, and the personal information of the patients have been discarded. An annotated/labeled corpus consisting of 500 discharge summaries and 492 progress notes has been manually created. The EMR data are written in Chinese with 55,485 sentences. The annotation was made by two Chinese physicians (A1 and A2) independently [7, 13]. It is categorized into five entity types: disease, symptom, treatment, test, and disease group. An annotation example is shown in Fig. 4. The character n-grams are conducted by word segmentation and named entity recognition on Chinese sentences. In the domain of natural language processing (NLP) on Chinese, the first step is to segment the sentence into words containing n-gram characters since for Chinese the minimum semantic units are words, not individual characters. It can be accomplished by NLP tools like Stanford Word Segmenter [20, 21]. Then for recognizing medical concepts from EMR, we define the named entity classes and use different labels to indicate these classes. For example, B/I/O labels denote the beginning word, inside word, and outside word of the named entities. Moreover, for named entity recognition on EMR, we attach the medical information to these three labels in order to denote different categories of named entities. For example, B_disease and B_treatment are denoting beginning words of disease and treatment named entities, respectively. The descriptions of entity types are given in Table 2.

**Table 2:** Name of the entity types and their descriptions

Entity Types	Description
Disease	Phrases related to disease concept
Symptom	Phrases of symptom concept
Disease group	Phrases of the cruelty of diseases
Treatment	Phrases of protocol and surgery name
Test	Phrases represent different tests name prescribed for patient

The categorized entity types are labeled in BIO format: B, starting of the medical entity type; I, inside of the medical entity type; O, apart from the entity type. The categorization of entities in BIO format is given in Table 3.

**Table 3:** BIO format of entity types

Categories							Total
NER type	Disease	Symptom	Disease group	Treatment	Test	Other	6
BIO format	B_dis	B_com	B_dit	B_tre	B_tes	other	11
	I_dis	I_com	I_dit	I_tre	I_tes		

### Experimental settings

In this experiment, our proposed model is employed to extract medical information from EMR dataset. The key hyper parameters are: Number of hidden neurons for each hidden layer: 150, Minibatch size: 20, Number of epoch: 100, Optimizer: Adam optimizer, Learning rate: 0.01, Learning rate decay: 0.9. They are determined by trial and error.

### Evaluation metric

Different metrics in terms of micro-average F score (MicroF), macro-average F score (MacroF) [22] and accuracy have been used to evaluate the performance of our proposed model. Accuracy is calculated by dividing the number of predicted entities that is exactly matched with dataset entities over the total number of entities in the dataset. MicroF is calculated by MicroP and MicroR values whereas MacroF is affected by the average *F* values of each class:

8 $$ F = \frac{2 \times P \times R}{P+R}.  $$

where *P* indicates precision measurement that defines the capability of a model to represent only related entities [23] and *R* (recall) computes the aptness to refer all corresponding entities:

9 $$ P= \frac{TP}{TP+FP}.  $$

10 $$ R= \frac{TP}{TP+FN}.  $$

whereas *TP* (True Positive) counts total number of entity matched with the entity in the labels. *FP* (False Positive) measures the number of recognized label does not match the annotated corpus dataset. *FN* (False Negative) counts the number of entity term that does not match the predicted label entity. Then,

11 $$ MacroF = \frac{1}{T} \sum\limits_{j=1}^{T} F_{j}.  $$

12 $$ MacroP= \frac{1}{T} \sum\limits_{j=1}^{T} P_{j}.  $$

13 $$ MacroR= \frac{1}{T} \sum\limits_{j=1}^{T} R_{j}.  $$

where *T* denotes the total number of categorized entities and *F*_*j*_, *P*_*j*_, *R*_*j*_ are *F*, *P*, *R* values in the *j*^*th*^ category of entities [7].

MicroP, MicroR, and MicroF are defined as following.

14 $$ MicroP = \frac{{\sum}_{j=1}^{T} {TP}_{j}}{{\sum}_{j=1}^{T} {TP}_{j} + {\sum}_{j=1}^{T} {FP}_{j}}.  $$

15 $$ MicroR = \frac{{\sum}_{j=1}^{T} {TP}_{j}}{{\sum}_{j=1}^{T} {TP}_{j} + {\sum}_{j=1}^{T} {FN}_{j}}.  $$

16 $$ MicroF = \frac{2 \times MicroP \times MicroR}{MicroP + MicroR}.  $$

### Experimental results

Our experiments are implemented in different phases namely micro average, macro average and accuracy comparison. Precision, Recall and F-score are measured using our proposed multitask bi-directional RNN model and compared with the following classifiers: Naive Bayes (NB), Maximum Entropy (ME), Support Vector Machine (SVM), Conditional Random Field (CRF) [7], and deep learning models including Convolutional Neural Network (CNN) [7], single task bi-directional RNN (Bi-RNN) and transfer bi-directional RNN [24], where NER can be defined as a multiclass classification problem for these classifiers [7]. Among all the models, we have considered Bi-RNN model as baseline model.

Firstly, performances are compared based on micro values and summarized in Tables 4 and 5. The results show that our proposed multitask bi-directional RNN model outperforms other models. For instance, the MicroF value of our proposed model is improved by 2.41% point and 4.67% point compared to the baseline model (Bi-RNN) and CNN, respectively in terms of results in Table 4. In addition, the MicroF value of our proposed model is improved by 3.07% point and 5.52% point compared to the baseline model (Bi-RNN) and CNN, respectively in terms of results in Table 5.

**Table 4:** Comparison results of MicroP, MicroR and MicroF measure on discharge summaries

Model	MicroP	MicroR	MicroF
Naive Bayes	78.07	77.91	77.99
Maximum Entropy	88.81	88.81	88.81
Support Vector Machine	90.52	90.52	90.52
Conditional Random Field [7]	93.15	93.15	93.15
Convolutional Neural Network [7]	88.64	88.64	88.64
Bi-RNN model	90.90	90.90	90.90
Transfer learning Bi-RNN model [24]	92.25	92.25	92.25
Our proposed model	93.31	93.31	93.31

**Table 5:** Comparison results of MicroP, MicroR and MicroF measure on progress notes

Model	MicroP	MicroR	MicroF
Naive Bayes	79.42	79.37	79.40
Maximum Entropy	91.45	91.45	91.45
Support Vector Machine	93.07	93.06	93.06
Conditional Random Field [7]	94.93	94.02	94.02
Convolutional Neural Network [7]	91.13	91.14	91.13
Bi-RNN model	93.58	93.58	93.58
Transfer learning Bi-RNN model [24]	94.37	94.37	94.37
Our proposed model	96.65	96.65	96.65

Since micro average only measures the effectiveness of model on a large number of entity, macro average is computed to evaluate the model’s performance in the case of small number of entity terms [25]. Table 6 shows the comparison results of NER on discharge summaries. The macro average F-score is improved by 4.16% point compared to the baseline model. The F-measure ranged from 57.14% point to 88.61% point in different categorized entities when it is computed on our proposed model whereas the range is from 54.54% point to 84.68% point when it is computed from the baseline model. Table 7 shows the comparison results of NER on progress note. The macro average F-score is improved by 13.82% compared to the baseline model. The F-measure ranged from 79.06% point to 94.56% point in different categorized entities when it is computed on our proposed model whereas the range is from 40.00% point to 89.52% point when it is computed from the baseline model.

**Table 6:** Comparison results of NER on discharge summaries

	Bi-RNN model			Our proposed model		
Entity type	Precision	Recall	F-measure	Precision	Recall	F-measure
Disease	82.82	78.02	80.34	84.11	84.70	84.40
Symptom	80.26	80.11	80.19	88.08	84.01	86.00
Disease group	37.50	100	54.54	43.75	82.35	57.14
Treatment	68.89	78.58	73.41	73.91	82.06	77.77
Test	82.99	86.43	84.68	89.23	87.99	88.61
Macro average	70.91	84.67	74.63	75.82	84.22	78.79

**Table 7:** Comparison results of NER on progress notes

	Bi-RNN model			Our proposed model		
Entity type	Precision	Recall	F-measure	Precision	Recall	F-measure
Disease	90.11	88.93	89.52	94.06	95.07	94.56
Symptom	87.67	88.335	88.00	94.50	90.79	92.61
Disease group	27.27	75.00	40.00	77.27	80.95	79.06
Treatment	71.06	77.80	74.28	88.15	87.19	87.67
Test	83.64	88.41	85.96	92.53	93.36	92.94
Macro average	71.95	83.69	75.55	89.31	89.47	89.37

The comparison results of accuracy on discharge summaries and progress notes are given in Tables 8 and 9. It is observed that the overall accuracy is improved by 5.66% point and 9.41% point on discharge summary and progress note, respectively, compared to the baseline model. According to the evaluation results, our proposed model shows better performance on recognizing medical entity terms comparing with other models including CRF model. CRF uses the feature templates to extract features in order to build the NER model by introducing prior knowledge. On the other hand, the proposed model performs the NER task on Chinese EMRs without any prior knowledge.

**Table 8:** Comparison results (%accuracy) on discharge summaries

Model	Entity type					
	Disease	Symptom	Disease group	Treatment	Test	Overall accuracy
Naive Bayes (NB)	44.82	51.72	N/A	59.00	65.96	58.91
Maximum Entropy (ME)	48.32	56.34	34.19	58.80	76.10	65.68
Support Vector Machine (SVM)	57.18	62.52	37.22	60.48	80.17	70.46
Conditional Random Field (CRF) [7]	77.33	77.83	48.39	77.47	90.05	83.94
Convolutional Neural Network(CNN) [7]	52.80	65.76	40.00	53.14	79.28	68.60
Bi-RNN model	73.83	79.35	28.00	67.99	82.63	77.85
Transfer learning Bi-RNN model [24]	74.30	82.60	44.00	68.20	86.79	80.75
Our proposed model	76.86	87.22	36.00	71.33	89.20	83.51

**Table 9:** Comparison results (%accuracy) on progress notes

Model	Entity type					
	Disease	Symptom	Disease group	Treatment	Test	Overall accuracy
Naive Bayes (NB)	69.50	70.09	N/A	41.59	71.85	67.49
Maximum Entropy (ME)	71.49	72.37	41.15	52.93	77.58	72.44
Support Vector Machine (SVM)	77.77	76.92	21.12	56.36	81.49	76.45
Conditional Random Field (CRF) [7]	87.42	87.09	36.06	75.60	90.31	87.22
Convolutional Neural Network(CNN) [7]	76.19	76.65	12.50	51.83	76.65	73.40
Bi-RNN model	87.48	87.01	25.00	63.99	83.75	82.72
Transfer learning Bi-RNN model [24]	88.70	88.49	31.25	72.93	86.12	85.43
Our proposed model	92.24	94.19	75.00	86.46	92.61	92.13

It is observed that the best accuracy is enlisted as 89.20% point in test terms and lowest performance is 36.00% point in recognizing disease terms for the case of discharge summary. The accuracy of recognizing disease terms is lowest comparing with other entities since there are very limited number of disease group (0.56% point) [24] in sample which is not enough to train the model. Similar observations are gained for the case of progress note.

In addition, we examine how different features affect the model performance on the discharge summary data. We compare the proposed models built by word level features, character level features, and combined word level features and character level features. The comparison results are shown in Table 10. It is observed that combined features improve the model performance.

**Table 10:** Comparison results for character and word level feature

Embedding approaches	Character level	Word level	Character level+Word level
MicroF	77.25	93.22	93.31
MacroF	47.28	81.23	78.79
Accuracy	35.30	83.12	83.51

## Discussion

In our proposed multitask model, we have been concentrating on improving the accuracy of named entity recognition task. Therefore, we have used different task layer (parts-of-speech tagging task) to enhance recognition performance which in turn improves the accuracy of named entity recognition task. More training time is needed for the proposed model since two task specific layers need to be trained, which involves two loss functions and two optimizers. We plan to use a joint loss function and joint optimizer to reduce the training time and improve the accuracy in our future research.

## Conclusions

In this paper, a novel multitask bi-directional RNN model is proposed for improving the performance of named entity recognition in EMR. Two different task layers, namely parts of speech tagging task layer and named entity recognition task layer are used in order to improve the information extraction method from EMR dataset by sharing the word embedding and character embedding layer. The feature sharing layer has a great impact on improving the accuracy of extracting entity information. Evaluation results using real datasets demonstrate the effectiveness of the proposed model.

## Abbreviations

CNN: Convolutional neural network
CRF: Conditional random field
EMR: Electronic medical record
FN: False negative
FP: False positive
LSTM: Long short-term memory
ME: Maximum entropy
NB: Naive Bayes
NER: Named entity recognition
NLP: Natural language processing
RNN: Recurrent neural network
SVM: Support vector machine
TP: True positive
